# Cystatin C and α-1-Microglobulin Predict Severe Acute Kidney Injury in Patients with Hemorrhagic Fever with Renal Syndrome

**DOI:** 10.3390/pathogens9080666

**Published:** 2020-08-18

**Authors:** Magnus Hansson, Rasmus Gustafsson, Chloé Jacquet, Nedia Chebaane, Simon Satchell, Therese Thunberg, Clas Ahlm, Anne-Marie Fors Connolly

**Affiliations:** 1 Clinical Chemistry, Karolinska University Hospital, 17176 Stockholm, Sweden; magnus.d.hansson@sll.se; 2 Department of Laboratory Medicine, Karolinska Institutet, 17177 Stockholm, Sweden; 3 Department of Clinical Neuroscience, Karolinska Institutet, 17177 Stockholm, Sweden; Rasmus.Gustafsson@ki.se; 4 Department of Clinical Microbiology, Umeå University, 90185 Umeå, Sweden; chloe.jacquet@umu.se (C.J.); nedia.chebaane@hotmail.com (N.C.); therese.thunberg@umu.se (T.T.); clas.ahlm@umu.se (C.A.); 5 Molecular Infection Medicine Sweden (MIMS), Umeå University, 90187 Umeå, Sweden; 6 Bristol Renal, Bristol Medical School, University of Bristol, Bristol BS1 3NY, UK; s.c.satchell@bristol.ac.uk

**Keywords:** acute kidney injury, hemorrhagic fever with renal syndrome, orthohantavirus, puumala virus, viral hemorrhagic fever, cystatin C, α-1-microglobulin, neutrophil gelatinase-associated lipocalin, creatinine

## Abstract

Puumala orthohantavirus causes hemorrhagic fever with renal syndrome (HFRS) characterized by acute kidney injury (AKI), an abrupt decrease in renal function. Creatinine is routinely used to detect and quantify AKI; however, early AKI may not be reflected in increased creatinine levels. Therefore, kidney injury markers that can predict AKI are needed. The potential of the kidney injury markers urea, cystatin C, α1-microglobulin (A1M) and neutrophil gelatinase-associated lipocalin (NGAL) to detect early AKI during HFRS was studied by quantifying the levels of these markers in consecutively obtained plasma (P) and urine samples (U) for 44 HFRS patients. P-cystatin C and U-A1M levels were significantly increased during early HFRS compared to follow-up. In a receiver operating characteristic (ROC) curve analysis, P-cystatin C, U-A1M and P-urea predicted severe AKI with area under the curve 0.72, 0.73 and 0.71, respectively, whereas the traditional kidney injury biomarkers creatinine and U-albumin did not predict AKI. Nearly half of the HFRS patients (41%) fulfilled the criteria for shrunken pore syndrome, which was associated with the level of inflammation as measured by P-CRP. P-cystatin C and U-A1M are more sensitive and earlier markers compared to creatinine in predicting kidney injury during HFRS.

## 1. Introduction

Orthohantaviruses (henceforth termed hantaviruses) can cause the viral hemorrhagic fever diseases: hemorrhagic fever with renal syndrome (HFRS) or hantavirus cardiopulmonary syndrome [1,2]. These diseases are characterized by fever, inflammation, capillary leakage, acute kidney injury (AKI) and lung damage. Studies have shown that these diseases may be more similar than previously assumed, in that HFRS patients have respiratory symptoms and lung damage [3,4]. HFRS caused by Puumala hantavirus (PUUV) has a mortality rate of 0.4% [5,6]. AKI is a common complication during HFRS that can range from being asymptomatic to anuria [2,7]. Patients with severe AKI require longer hospitalization and sometimes hemodialysis. It is important to assess and predict the impaired kidney function during HFRS to ensure correct adjustment of ongoing medication that is excreted via the kidneys to avoid side-effects and intoxication. Kidney function is measured as decreased glomerular filtration rate (GFR) using plasma creatinine levels, the volume of urine per day and also by immunohistochemical analysis of kidney biopsy samples [8,9]. Kidney biopsies from HFRS patients have shown presence of acute tubulointerstitial nephritis [10]. The mechanisms of renal dysfunction during PUUV infection are however poorly understood.

AKI is stratified into the severity stages 1, 2 and 3 based on the guidelines recommended by KDIGO 2012 (Kidney Disease: Improving Global Outcomes) using primarily plasma creatinine levels [9]. Unfortunately, creatinine levels are not particularly sensitive for measuring AKI since GFR has to decrease by approximately 50% before creatinine levels increase [11,12]. Furthermore, serum creatinine levels correlate with muscle mass and meat consumption, which may result in over- or underestimation of GFR [13]. New biomarkers to improve early diagnosis of AKI have been evaluated previously including cystatin C, α-1-microglobulin (A1M) and neutrophil gelatinase-associated lipocalin (NGAL). Cystatin C is a cysteine-proteinase inhibitor and is found in all nucleated cells [14]. It is normally filtrated from the blood stream by glomerular filtration, followed by resorption and metabolization in proximal renal tubules, which explains the elevated Cystatin C levels in plasma with declining GFR [15]. Cystatin C is less influenced by age and not affected by sex, muscle mass or diet, compared to plasma creatinine [16,17,18], and has previously been shown to be superior to creatinine in indicating renal impairment [19]. A1M is a 27 kDa glycoprotein that is reabsorbed by renal tubular cells, and following tubular injury, the levels of urinary (U)-A1M increase [20]. NGAL is a 25 kDa sized protein and is expressed in neutrophils and kidney epithelial cells among others. It is freely filtered in the glomerulus and reabsorbed by tubular cells as A1M [21]. In previous studies of kidney injury during HFRS, the levels of U-NGAL, U-A1M and P-Cystatin C were increased in patients with AKI, indicating their potential as kidney damage biomarkers [22,23,24,25,26].

Another kidney disorder, the shrunken pore syndrome (SPS) describes the impairment of glomerular filtration of molecular sizes between 12–29 kDa [27,28]. SPS is defined by the ratio of eGFR calculated using cystatin C over eGFR calculated using creatinine, and if the ratio is less than 0.7, the criteria are fulfilled [29]. In previous studies, the 3- and 5-year mortality was shown to increase in patients which fulfilled the SPS criteria [29]. To the best of our knowledge, SPS has not been studied in patients with acute infectious diseases. 

Hence, the main aims of our study were to determine which kidney injury markers increased during early HFRS, to identify which of them could predict severe AKI in HFRS patients, and to provide a descriptive study to determine whether SPS could be present during HFRS. 

## 2. Results

### 2.1. Characteristics of HFRS Patients Included in the Study

For all 44 patients, demography, hospital care characteristics and laboratory values are shown in Table 1. The patients were stratified into groups of whether they fulfilled criteria for AKI stage 1, 2 or 3 or no AKI at any time point during HFRS. Furthermore, patients were stratified into groups of no severe AKI (no AKI or stage 1) and severe AKI (stage 2 or 3) based on KDIGO criteria. As described in Table 1, 17 (38.6%) patients had no severe AKI and 27 (61.4%) patients developed severe AKI. For 5 patients, urine samples were missing. The majority of patients required hospitalization (n = 36; 82%). In addition, 14 HFRS patients had severe AKI at first sample and were therefore excluded in the receiver operating characteristic (ROC) curve analysis for prediction of severe AKI. The criteria for SPS was fulfilled for 41% (18/44) HFRS patients; however, this was transient in all but one patient. None of the HFRS patients had kidney disorders prior to infection. Three of the patients had rheumatic disorder prior to HFRS; therefore, corticosteroid treatment could potentially have been prescribed, which may bias the data. However, information on potential medication the patients received prior to HFRS was not available. Apart from these, no other patients had conditions potentially requiring corticosteroid treatment.

### 2.2. P-Cystatin C and U-A1M Levels Increase Earlier than P-Urea and P-Creatine during HFRS

**Figure 1:**
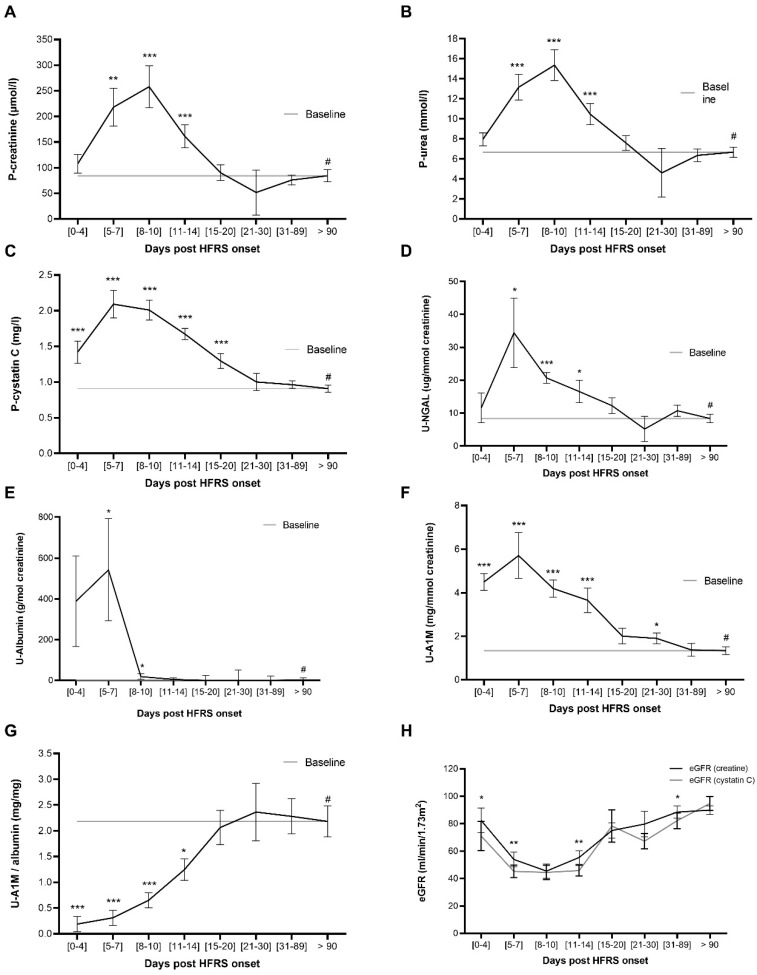
Timeline kinetics of kidney injury markers in HFRS patient plasma and urine. The plasma levels of creatinine (**A**), urea (**B**) and cystatin C (**C**) and the urinary levels of NGAL (**D**), albumin (**E**), A1M (**F**) and ratio of A1M and albumin (**G**) were quantified in patient plasma obtained at different time points and the estimated mean and standard error of the mean calculated using the generalized estimating equation (GEE) method and compared to follow-up at least 90 days post disease onset using the GEE method. Furthermore, the estimated glomerular filtration rate (eGFR) was calculated based on creatinine (CKD-EPI) or Cystatin C (CAPA) using the GEE method, and differences between eGFR for each time point were calculated using Wilcoxon signed rank test for paired samples (**H**). Significant differences between the time point and follow-up (#: >90 days post onset of HFRS) (A–G) or between eGFR within the same time point (**H**) are indicated by asterisks (*** *p* < 0.001; ** *p* < 0.01; * *p* < 0.05). A1M: α-1 microglobulin; CAPA: Caucasian and Asian Pediatric and adult subjects; CKD-EPI: chronic kidney disease epidemiology collaboration; eGFR: estimated glomerular filtration rate; HFRS: hemorrhagic fever with renal syndrome; P: plasma; NGAL: neutrophil gelatinase-associated lipocalin; U: urine.

The levels of study (P-cystatin C, U-A1M and U-NGAL) and conventional (P-creatinine, P-urea and U-albumin) kidney injury markers in patient plasma and urine were quantified and the kinetics visualized in Figure 1. The aim was to identify at which time points the kidney damage levels were significantly increased compared to follow-up (>90 days post disease onset; DPDO), which was used as the base line since the AKI caused by HFRS is transient. P-cystatin C (Figure 1C) and U-A1M (Figure 1F) are significantly increased at the earliest time point (0–4 DPDO) compared to follow-up (<90 DPDO). P-creatinine (Figure 1A), P-urea (Figure 1B), U-NGAL (Figure 1D) and U-albumin (Figure 1E) also increase significantly, however, not before 5–7 DPDO. All studied kidney injury markers return to base line within the first three weeks following HFRS onset (Figure 1A–F). 

To determine whether estimated glomerular filtration rate (eGFR) calculated using P-cystatin C is lower compared to eGFR calculated using creatinine, the eGFR was quantified and the kinetics for both eGFR compared in Figure 1H. The eGFR_cystatin c_ was significantly lower than eGFR_creatinine_ in the first week following HFRS onset (0–4 and 5–7 DPDO). Furthermore, eGFR_cystatin c_ decreases significantly in the earliest time point (0–4 DPDO) compared to follow-up (<90 DPDO) (Figure S1), reflecting the earlier increase in P-cystatin C as shown in Figure 1C. As for P-creatinine, the eGFR_creatinine_ also significantly decreased compared to follow-up, but first from 5–7 DPDO (Figure S1). 

A potential confounder is corticosteroid treatment since it can increase cystatin C levels without presence of renal function impairment [30]. To study whether potential corticosteroid treatment could be a confounder in our studies, the mean levels of maximum cystatin C were determined and compared between the three patients who might have received corticosteroid treatment compared to the rest of the HFRS cohort. The mean level was 2.2 (standard deviation (SD) = 1) in the three patients with rheumatic disorders and 2.3 (SD = 1.2) in the remaining HFRS patients. Using the non-parametric Mann–Whitney U-test, there was no significant difference in the maximum level of cystatin C between these patient groups. In addition, when adjusting for potential need of corticosteroid treatment in the GEE model for cystatin C kinetics, there were no significant interactions. Thereby, corticosteroid treatment does not appear to be a confounder in our study.

### 2.3. P-Cystatin C, U-A1M and U-NGAL Levels are Significantly Increased in HFRS Patients with Severe AKI

**Figure 2:**
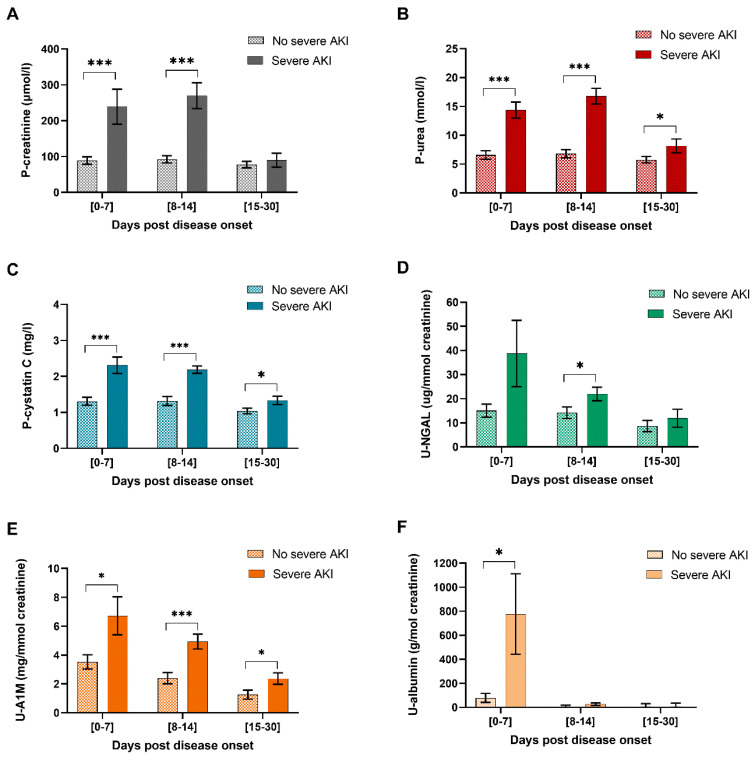
Kidney injury marker levels stratified across HFRS patients with severe AKI. HFRS patients were stratified into two groups of severe AKI (stage 2/3) or not (AKI stage 0/1) according to KDIGO criteria 2012. The estimated mean and standard error of the kidney injury markers for each group were calculated using generalized estimating equation (GEE) and adjusted for sex and age. The timeline kinetics are shown for P-Creatinine (**A**), P-urea (**B**), P-cystatin C (**C**), U-NGAL (**D**), U-A1M (**E**) and U-albumin (**F**). Significant differences within the same time point between kidney injury marker levels in HFRS patients stratified across no severe AKI vs. severe AKI are indicated by asterisks (*** *p* < 0.001; **p < 0.01; * *p* < 0.05). A1M: U-α-1 microglobulin; AKI: Acute kidney injury; HFRS: hemorrhagic fever with renal syndrome; P: plasma NGAL: neutrophil gelatinase-associated lipocalin; U: urine.

To determine whether the study and conventional kidney injury markers are increased in patients with severe AKI (stage 2 or 3) compared to HFRS patients that did not fulfil these criteria, the levels of kidney injury markers were stratified into different time groups and compared between the two groups within each time group (Figure 2). AKI stratification is based on P-creatinine, and thereby, these levels will be increased in severe AKI patients but were included in Figure 2 to enable comparison with the other kidney injury markers. In order to ensure that all time points are represented by as many patients as possible for the two severe AKI stratification groups, the time groups were expanded to 0–7; 8–14 and 15–30 DPDO, respectively. The conventional kidney injury markers, P-creatinine, P-urea and U-albumin were increased in HFRS patients with severe AKI (Figure 2A,B,F). The studied kidney injury markers P-cystatin C (Figure 2C) and U-A1M (Figure 2E), were increased in patients with severe AKI throughout HFRS, apart from U-NGAL, which was only significantly increased at days 8–14 post HFRS onset (Figure 2D). Thereby, the studied kidney injury markers, P-cystatin C, U-A1M and U-NGAL, were significantly increased in HFRS patients with severe AKI, verifying previous studies [19,24,31] and ascertaining their use as markers of kidney injury. 

### 2.4. P-Cystatin C Levels Associate Significantly with the Levels of the Traditional Kidney Injury Markers P-Creatinine and P-Urea during HFRS

To investigate whether the studied kidney injury markers, P-cystatin C, U-A1M and U-NGAL, associate significantly with the traditional kidney injury markers P-creatinine and P-urea, all plasma and urine samples for all patients obtained during HFRS (≤30 DPDO) were analyzed using the GEE method and adjusted for sex and age (Table 2). P-cystatin C associated significantly with the conventional kidney injury markers P-creatinine and P-urea. U-A1M only associated significantly with P-creatinine; and U-NGAL did not associate with either of P-creatinine or P-urea. These results support the role for P-cystatin C as a kidney injury marker during HFRS. However, when only the peak levels of the kidney injury markers were included, P-cystatin C, U-A1M and U-NGAL associated significantly with P-creatinine and P-urea (Table S1). This analysis was included in order to enable comparison with results obtained in another study in our discussion [25].

### 2.5. P-Cystatin C, U-A1M and P-Urea Predict Severe AKI in a ROC Curve Analysis

The ability of the studied kidney injury markers (P-cystatin C, U-A1M and U-NGAL) vs. traditional kidney injury markers (P-creatinine, P-urea and U-albumin) to predict severe AKI and the following cut-off level were determined using a ROC curve analysis (Table 3). In order to study only future AKI, patients who had severe AKI at first sample were excluded; thereby, there were 30 HFRS patients to perform our ROC curve analysis. The traditional kidney injury markers P-creatinine and U-albumin did not predict severe AKI (Table 3) during the later phase of HFRS whereas P-urea, P-cystatin C and U-A1M could predict severe AKI, with similar AUC. U-NGAL levels in the first sample were significant but instead of the highest level predicting severe AKI, it seemed that high levels predicted no severe AKI, which is contrary to expectation. The corresponding ROC curves are shown in Figure S2. 

### 2.6. Shrunken Pore Syndrome in HFRS Patients

To determine whether there is a pathophysiological association between SPS and disease outcome, the maximum levels of the kidney injury markers, P-cystatin C, P-urea, P-creatinine, U-A1M and U-NGAL, and CRP, a marker of inflammation, were analyzed for significant differences between patients who fulfilled SPS criteria at any time point during HFRS vs. those who did not. None of the studied kidney damage markers were increased in patients who fulfilled SPS criteria during HFRS (Table 4), nor did patients who fulfilled SPS criteria require longer hospitalization. Though not significant, the patients who fulfilled SPS criteria were older (Table 4). However, CRP levels were increased in HFRS patients fulfilling SPS criteria indicating an association between inflammation and SPS (Table 4). More precisely, the CRP levels of SPS patients were increased for the time groups 0–7 DPDO and 15–30 DPDO (Figure S3).

## 3. Discussion

AKI is a common feature during HFRS [2], and in our study, we show that P-cystatin C, U-A1M and P-urea could predict severe AKI, which P-creatinine and U-albumin failed to do. Furthermore, both P-cystatin C and U-A1M levels increased earlier than the traditional kidney injury markers creatinine, urea, and U-albumin in all HFRS patients. In addition, eGFR_cystatin C_ decreased significantly compared to eGFR_creatinine_ during early HFRS. We also show that nearly half (41%) of the patients fulfilled criteria for SPS during HFRS. To the best of our knowledge, this is the first time SPS has been studied in a cohort of infectious disease patients.

According to the KDIGO 2012 guidelines, AKI severity is defined primarily by increased levels of creatinine in patient plasma [9]. However, creatinine levels do not increase before kidney function as measured by eGFR is decreased by 50%, with the risk of missing early kidney damage [11,12]. Early detection of kidney damage is crucial as important medication that is excreted via the kidneys could be impaired if eGFR is decreased, and thereby, an insufficient effect of the medication may be achieved. We show that P-cystatin C, U-A1M and P-urea are more sensitive markers of kidney damage compared to the traditional kidney injury marker P-creatinine. This verifies earlier findings that P-cystatin C and U-A1M are increased in patients infected with hantaviruses [24,31,32]. In a study of 32 patients infected with Hantaan virus, the prototype hantavirus, the levels of cystatin C in serum increased to peak during the oliguric phase, to then normalize, which is similar to our findings. In addition, the levels of cystatin C in serum samples from patients infected with Hantaan virus were significantly elevated in the earliest disease phase (febrile/hypotensive) compared to serum cystatin C levels in serum samples from control individuals. Serum creatinine levels did not differ between Hantaan virus infected patients compared to control individuals in the earliest disease phase, indicating that cystatin C is more sensitive in detecting renal function in the earliest stages of disease [24]. In our study, we compare the levels of cystatin C and creatinine in plasma samples from PUUV-infected patients during disease with follow-up; thereby, the patients become their own controls, and we avoid any potential bias or confounders that may occur when comparing to control individuals. We found that cystatin C levels in plasma significantly increased in the earliest phase of disease compared to follow-up, whereas creatinine levels did not differ significantly from follow-up in the earliest phase. Though we compare the levels of cystatin C and creatinine within the PUUV-infected patients included in our study, we find similar results to the study performed by Ma et al., thereby, verifying that infection with hantaviruses (Hantaan or PUUV) leads to earlier increase of cystatin C levels than creatinine, indicating cystatin C is indeed a more sensitive kidney injury marker than creatinine. Furthermore, Ma et al., found that the ratio of urinary cystatin C levels in urine samples obtained within 10 days of fever onset compared to the levels of urinary cystatin C in samples from control individuals increased significantly between disease severity stages low, mild and high according to Chinese diagnostic disease severity criteria [24]. In our study, we use KDIGO 2012 criteria for acute kidney injury, and using ROC curve analysis, we find that the plasma cystatin C level obtained in the first sample could predict severe AKI (stage 2 or 3 according to KDIGO 2012). Thereby, though we use different severity outcomes compared to Ma et al., we find that cystatin C levels can be used as a prognostic factor for hantavirus disease outcome. In a study of patients infected with Dobrova-Belgrade hantavirus, the patients in need of hemodialysis had significantly higher levels of U-A1M levels compared to those who did not need hemodialysis [32]. Though none of our PUUV-infected patients required hemodialysis, U-A1M levels in the first sample could predict severe AKI (as defined by KDIGO 2012 criteria), supporting the finding that U-A1M levels associate with disease outcome following infection with either PUUV or Dobrova-Belgrade hantavirus. The underlying mechanisms of kidney damage during HFRS are not yet clear; however, renal glomerular and tubular cells can be infected with PUUV resulting in functional alterations as shown in kidney biopsies from PUUV infected patients and in vitro [33,34]. This might explain the findings in our study with increased kidney injury markers in HFRS patients. In other studies of patients with a variety of disorders ranging from need of cardiac surgery, prior kidney diseases, sepsis and patients in the intensive care unit, P-cystatin C proved to be an earlier predictor of AKI [35,36,37,38,39], and our data supports these findings. One potential confounder for cystatin C levels is treatment with corticosteroids. In the present study, corticosteroid treatment did not seem to be an interacting factor, since the patients that could potentially have been prescribed corticosteroid treatment for their rheumatic disorder did not have higher levels of cystatin C in their plasma samples. In addition, HFRS patients do not receive corticosteroid treatment. 

The levels of U-NGAL in the present study were increased in HFRS patients with severe AKI compared to HFRS patients with no severe AKI, but U-NGAL levels did not increase during early HFRS. Furthermore, U-NGAL did not predict severe AKI in the present study. A previous study reports data on HFRS caused by PUUV where the peak level of U-NGAL associated significantly with the peak level of creatinine, indicating a pathophysiological association [25]. Even though we found a similar result in our study, our study does not support U-NGAL as an early predictive marker of severe AKI. 

Nearly half (41%) of the HFRS patients fulfilled the criteria for SPS, and the levels of CRP, a marker of inflammation, were increased; however, these patients did not have increased kidney damage marker levels. In several previous studies, SPS has been shown to be associated with increased mortality and morbidity [29]. SPS is a well-documented predictor for poor clinical prognosis with an increase in the all-cause mortality for those who fulfilled SPS criteria at first visit in a 10-year longitudinal study, even in the absence of albuminuria [40]. In another study, patients in need of cardiac surgery had higher 1- and 3-year mortality if they had SPS at the time of operation [41]. The underlying mechanism for the observed increased long-term mortality and morbidity has been proposed to be due to increased levels of proteins that facilitate atherosclerosis [42]. Our study is a short-term study, where transient increase of kidney damage markers during an acute viral infection are investigated. It is interesting to note that nearly half of the patients fulfill the criteria for SPS, but what this means in the long-term in terms of mortality or morbidity remains to be investigated. 

In conclusion, we show that P-urea, P-cystatin C and U-A1M predicts future severe AKI during HFRS. In addition, P-cystatin C and U-A1M levels increase significantly during early time points compared to follow-up, which P-creatinine did not. We therefore highlight the potential of P-cystatin C and U-A1M as early and sensitive markers of kidney injury during HFRS and likely also for other diseases.

## 4. Materials and Methods

### 4.1. Study Cohort

In total, 44 patients were enrolled in this study after being diagnosed with PUUV infection during 2008–2014 at the Infectious Disease Clinic, Umeå University hospital. The diagnosis of PUUV infection was verified by the patient having typical clinical manifestations of HFRS and presence of immunoglobulin (Ig)G and IgM antibodies towards PUUV detected using an immunofluorescence assay. To be included in the study, the patients had to give oral and written informed consent. Following inclusion, blood and urine samples were collected at different time points during HFRS and ending with a follow-up sample at least 90 DPDO. The peripheral venous blood samples were collected using commercially available vacutainers containing sodium heparin as the anti-coagulant (Becton Dickinson, Franklin Lakes, NJ, US). The vacutainers were centrifuged and plasma aliquoted into different vials immediately stored at −80 °C until use. Urine was collected and frozen at −80 °C for later analyses.

### 4.2. Routine Clinically Laboratory Analyses

Blood and urine samples from patients were analyzed at the accredited Clinical Chemistry Laboratory, University Hospital of Umeå, for levels of P-creatine, P-cystatin-C, P-urea, P-CRP, U-albumin and U-creatinine. The concentration of U-albumin was normalized using urinary creatinine concentration.

### 4.3. Kidney Injury Markers

The levels of U-NGAL and U-A1M were quantified in urine samples from HFRS patients obtained at different time points during HFRS and at follow-up, at the central accredited Clinical Chemistry Laboratory at the Karolinska University hospital. U-NGAL was measured using a Cobas^®^ 8000 c502 instrument (Roche Diagnostics, Rotkreutz, Switzerland) with particle-enhanced turbidimetric immunoassay reagents (Gentian AS, Moss, Norway). U-A1M was measured using a BN ProSpec instrument (Siemens Healthcare, Erlangen, Germany) by immune nephelometry with reagents from Siemens. The samples were run in batches of 30–50 samples at a time, and control samples were analyzed prior to analysis of each batch of samples. The same lots of reagents, calibrators and controls were used for both U-NGAL and U-A1M throughout the study. The concentrations of U-NGAL and U-A1M were normalized using urinary creatinine concentration. 

### 4.4. Severity of Acute Kidney Injury

The severity of AKI during HFRS was scored according to KDIGO 2012 guidelines [9] into no AKI or AKI stage 1 to 3 based on the following criteria. P-creatinine (μmol/L) increased compared to baseline P-creatine levels: Stage 1, more than 1.5-fold; Stage 2, more than 2-fold; or Stage 3, more than 3-fold from baseline or above 353.6 μmol/L. The baseline P-creatinine was obtained for each patient if information regarding creatinine existed prior to HFRS or after follow-up when P-creatinine had normalized.

We define the outcome “severe AKI” as the patients who fulfilled AKI stage 2 or 3 at any time during HFRS (≤30 DPDO). 

### 4.5. Glomerular Filtration Rate and Shrunken Pore Syndrome

The eGFR was calculated with the Chronic Kidney Disease Epidemiology Collaboration (CDK-EPI) equations based on P-creatinine levels and sex. For women, the equations were as follows: P-creatinine < 62 µmol/L: 144 × (Scr/0.7)^−0.329^ × (0.993)^Age^ and if P-creatinine > 62 µmol/L: 144 × (Scr/0.7)^−1.209^ × (0.993)^Age^. For men, the equations were as follows: P-creatinine < 80 µmol/L: 141 × (Scr/0.9)^−0.411^ × (0.993)^Age^ and if P-creatinine > 80 µmol/L: 141 × (Scr/0.9)^−1.209^ × (0.993)^Age^ [43].

In order to determine whether patients fulfilled shrunken pore syndrome, eGFR was also calculated using the revised Lund–Malmö (eGFR LM-rev) equations based on P-creatinine levels and sex. For women if P-creatinine < 150 µmol/L: e^((2.5 + (0.0121 × (150 − creatinine))) − (0.0158 × age) + (0.438 × Ln(age))), and if P-creatine ≥ 150 µmol/L e^((2.5 − (0.926 × Ln(creatinine/150))) − (0.0158 × age)+(0.438 × Ln(age))). For men if P-creatinine < 180 µmol/L: e^((2.56 + (0.00968 × (180 − creatinine))) − (0.0158 × age) + (0.438 × Ln(age))) and if P-creatinine ≥ 180 µmol/L e^((2.56 − (0.926 × Ln(creatinine/180))) − (0.0158 × age) + (0.438 × Ln(age))) [27,28]. 

The eGFR was also calculated using P-cystatin C levels using the Caucasian and Asian pediatric and adult subjects (CAPA) formula: 130 × (cystatin C)^−1.069^ × (Age)^−0.117^ − 7 [44]. 

The criteria for Shrunken pore syndrome (SPS) was fulfilled if the ratio between eGFR calculated using cystatin C (CAPA) and creatinine (LM Rev) was below 0.7 [27,28].

### 4.6. Statistics

The generalized estimating equation (GEE) method with assumption of exchangeable correlation structure between consecutive observations was used to quantify the estimated mean and standard error of the mean (SEM) for longitudinal changes in the levels of kidney injury markers in plasma (creatinine, urea, cystatin C), urine (NGAL, A1M and albumin), eGFR calculated using either cystatin C or creatinine levels and the ratio of U-A1M to U-albumin. The estimated means within each specified time group (0–4; 5–7; 8–10; 11–14; 15–20; 21–30 and 31–89 DPDO) were compared to means at follow-up (at least 90 DPDO). Furthermore, whether eGFR_cystatin C_ differed to eGFR_creatinine_ within the same time point was determined using the Wilcoxon signed rank test for paired samples.

In order to visualize the difference in levels of kidney damage markers in HFRS patients with severe AKI compared to those who did not have severe AKI, the mean and standard error of the mean were calculated using the GEE method. Since the patients were stratified into two groups, the time points were extended to 0–7; 8–14 and 15–30 DPDO to ensure most patients had samples within the given time points. Whether the levels of kidney damage markers in plasma and urine differed between HFRS patient with severe AKI compared to those who did not have severe AKI was determined using the GEE method.

To determine the pathophysiological association between SPS and kidney damage markers, inflammation and whether patients with SPS were older or in increased need of hospital care, the generalized linear model was used. The estimated mean and standard error of the mean were calculated using the generalized linear model for HFRS patients stratified into whether they fulfilled SPS criteria at any time point during HFRS and tested for significant difference. Furthermore, the kinetics of kidney damage markers and marker of inflammation (CRP) were calculated using the GEE model and stratifying the patients into groups of those who fulfilled SPS criteria vs. those who did not. The difference within each time group between the two SPS groups was tested for significance using the GEE model.

In order to determine a potential pathophysiological association between the traditional kidney damage markers P-creatinine and P-urea and our studied kidney injury markers P-cystatin-C, U-NGAL and U-A1M was studied using the levels quantified in all plasma and urine samples during HFRS (≤30 DPDO) using the GEE method and adjusting for sex and age.

To determine whether the levels of the traditional kidney injury markers P-creatinine, P-urea and U-albumin, and our studied kidney injury markers P-cystatin C, U-A1M and U-NGAL can predict severe AKI, a ROC curve analysis was performed. Only samples obtained from HFRS patients that did not have severe AKI at time point of first sample collection were included in order to enable prediction of AKI. The cut-off value with highest sensitivity and specificity for each kidney damage marker was determined if area under the curve (AUC) was significant. 

The level of significance was set to *p* < 0.05. The statistical analyses were performed using SPSS version 25 (IBM, Armonk, NY, US).

### 4.7. Study Approval

The study was approved by the Ethical Committee of Umeå University (EPN 07-162M; amendment EPN 2014-37-32M). All patients were included after oral and written informed consent. 

## Figures and Tables

**Table 1:** Characteristics of the hemorrhagic fever with renal syndrome (HFRS) study group.

	HFRS Patientsn = 44 *	Reference Values
**Demographic data**		
Age, years	53 (41–64)	NA
Sex, n female/male (%)	29 (66%)/15 (34%)	NA
Hospital care, n (%)	36 (82%)	NA
Days of hospital care	6 (4–7.75)	NA
Day of first sample	6 (5–7)	NA
**Estimated glomerular filtration rate (eGFR) ^a^**		
eGFR—Creatinine (CKD-EPI) (mL/min/1.73 m^2^)	32 (15–67)	>60
eGFR—Creatinine (LM-Rev) (mL/min/1.73 m^2^)	28 (15–63)	>60
eGFR—Cystatin C (CAPA) (mL/min/1.73 m^2^)	27 (21–58)	>60
**Acute kidney injury (AKI) ^b^**		
No AKI	10 (22.7%)	NA
Stage 1	7 (15.9%)	NA
Stage 2	10 (22.7%)	NA
Stage 3	17 (38:6%)	NA
**Severe AKI ^c^**	27 (61.4%)	NA
**Shrunken Pore Syndrome ^d^**	18 (41%)	NA
**Kidney injury markers ^e^**		
Creatinine (μmol/L)	103 (65.75–163.5)	45–105
Urea (mmol/L)	8 (5–14.9)	2.6–8.1
Cystatin C (mg/L)	1.3 (0.98–2.28)	NA
U-A1M (mg/mmol creatinine)	4.4 (2.6–5.4)	NA
U-NGAL (µg/mmol creatinine)	16.4 (7.3–31)	NA
U-Albumin (g/mol creatinine)	47.4 (17.8–244.1)	<3

* Of the 44 HFRS patients, 5 were missing urine samples. Where the urinary concentration is calculated, it is based on urine samples obtained from 39 HFRS patients. ^a^ The estimated glomerular filtration rate (eGFR) was calculated using creatinine with the two different formulas CKD-EPI and LM-Revised, and for cystatin C using the CAPA formula. The median and interquartile range of the minimum eGFR observed at any time point during HFRS for eGFR calculated using the CKD-EPI, LM-rev and CAPA formula is shown. ^b^ Acute kidney injury (AKI) according to KDIGO guidelines 2012: Creatinine (μmol/L) increased more than 1.5-fold or increased more than 26.5 μmol/L within 48 h (stage 1); increased more than 2-fold from baseline (stage 2) or increased more than 3-fold from baseline or above 353.6 μmol/L (stage 3). ^c^ Severe AKI is defined as AKI stage 2 or 3 during HFRS. ^d^ Shrunken pore syndrome (SPS) is defined as the ratio between eGFR calculated using cystatin C (CAPA) and eGFR calculated using creatinine (LM-rev) being below 0.7. The number of patients that at any time point during HFRS fulfilled the criteria for SPS is shown. ^e^ Levels of kidney injury markers for all patients at day of first cystatin C sample, shown as median (interquartile range in brackets). HFRS: Hemorrhagic fever with renal syndrome; eGFR estimated glomerular filtration rate; AKI: acute kidney injury; NA: Not applicable; CKD-EPI: Chronic kidney disease epidemiology collaboration; LM-Rev: Lund-Malmö revised; CAPA: Caucasian and Asian Pediatric and Adult subjects; KDIGO: Kidney Disease Improving Global Outcomes; crea: creatinine; P: plasma; NGAL: Neutrophil gelatinase-associated lipocalin; U: urine.

**Table 2:** Association between kidney damage markers and renal dysfunction during HFRS ^a^.

Kidney Injury Markers	U-A1M (μg/mmol Creatinine)		U-NGAL (ng/mmol Creatinine)		P-cystatin C (mg/mL)	
	β	*p*-Value	β	*p*-Value	β	*p*-Value
P-Creatinine (μmol/L)	**0.01**	**0.036**	0.78	0.058	**150.4**	**<0.001**
P-Urea (nmol/L)	0.2	0.083	0.016	0.174	**6789**	**<0.001**

**^a^** Kidney injury markers (P-Creatinine and P-Urea) set as dependent variable. Only time points ≤ 30 days post disease onset included. The estimated β-coefficients were calculated using the generalized estimating equations method and shown with the corresponding *p* value. This corresponds to the change in the level of kidney dysfunction markers for 1 unit increase for kidney damage markers. Significant associations are shown highlighted in bold. All associations were adjusted for sex and age. A1M: α1-microglobulin; HFRS: Hemorrhagic fever with renal syndrome; NGAL: neutrophil gelatinase-associated lipocalin; P: plasma; U: urine.

**Table 3:** Prediction of severe AKI using ROC curve analysis of kidney injury markers ^a^.

Kidney Injury Markers	AUC	95% CI	*p*-Value	Cut-off Value	Sensitivity	Specificity
P-Urea (mmol/L)	**0.71**	**0.5–0.92**	**0.049**	**6.3**	**0.77**	**0.77**
P-Creatinine	0.65	0.45–0.85	0.17	**-**	**-**	**-**
U-Albumin (g/mol creatinine)	0.53	0.3–0.77	0.77	-	-	-
P-Cystatin C (mg/L)	**0.72**	**0.51–0.92**	**0.047**	**1.1**	**0.77**	**0.71**
U-A1M (mg/mmol creatinine)	**0.73**	**0.51–0.94**	**0.048**	**3.5**	**0.82**	**0.69**
U-NGAL (µg/mmol creatinine)	**0.27**	**0.05–0.46**	**0.034**	-	-	-

^a^ Acute kidney injury (AKI) according to KDIGO guidelines 2012: Creatinine (μmol/L) increased more than 2-fold from baseline (stage 2) or increased more than 3-fold from baseline or above 353.6 μmol/L (stage 3). The ability of admission value of kidney injury markers in patients’ plasma or urine to predict acute kidney injury stage 2/3 (yes/no) during HFRS was performed using ROC curve analysis. Only HFRS patients that did not fulfil AKI stage 2/3 at first sample were included in order to enable prediction of future AKI (n = 30: no severe AKI n = 17; severe AKI n = 13). For three patients, urine samples were missing; therefore, here there were n = 11 with severe AKI and n = 16 with no severe AKI. Significant results are shown in bold. A1M: α1-microglobulin; AUC: area under the curve; CI: confidence intervals; ROC: receiver operating characteristics; NGAL: neutrophil gelatinase-associated lipocalin; P: plasma; U: urine.

**Table 4:** Shrunken pore syndrome in HFRS patients ^a^.

Kidney Injury Markers	No SPS (n = 26)	SPS (n = 18)	*p*-Value
P-Creatinine (μmol/L)	273 (45)	230 (56)	0.561
P-Urea (nmol/L)	14 (2)	16 (2)	0.499
P-Cystatin C (mg/L)	2 (0.2)	2.6 (0.3)	0.087
U-Albumin (g/mol crea)	859 (332)	110 (377)	0.136
U-A1M (mg/mmol crea)	7.2 (1.6)	5.8 (1.8)	0.572
U-NGAL (ng/mmol crea)	56.5 (14.8)	23 (17)	0.133
CRP (mg/L)	**79 (10)**	**125 (13)**	**0.005**
Hospitalization time (days)	5.6 (1.3)	8 (1.3)	0.209
Age (years)	48 (3)	56 (3)	0.065

^a^ The shrunken pore syndrome (SPS) is defined as the ratio between eGFR_cystatin C_ (CAPA) vs. eGFR_creatinine_ (LM-Rev) below 0.7. The patients are stratified into whether they fulfilled SPS criteria at any time point during HFRS. To determine the difference in levels of biomarkers for kidney injury or inflammation, difference in age or need for hospitalization, the estimated mean and corresponding standard error of the mean were calculated and tested for significant difference between patients fulfilling SPS during HFRS using the generalized linear model. Significant results are shown in bold. SPS: Shrunken pore syndrome; HFRS: Hemorrhagic fever with renal syndrome; CAPA: Caucasian and Asian pediatric and adult subjects: LM rev: Lund–Malmö revised formula; A1M: α1-microglobulin; NGAL: neutrophil gelatinase-associated lipocalin; CRP: C-reactive protein; P: plasma; U: urine.

## Supplementary Materials

The following are available online at https://www.mdpi.com/2076-0817/9/8/666/s1. Figure S1: The estimated glomerular filtration rate (eGFR) was calculated based on creatinine (CKD-EPI) or Cystatin C (CAPA). The estimated mean and standard error of the markers and eGFR were calculated using generalized estimating equation (GEE) and are adjusted for sex and age. Significant difference between the time point and follow-up (#: >90 days post onset of HFRS) is indicated by asterisks (*** *p* < 0.001; ** *p* < 0.01; * *p* < 0.05). Figure S2: Receiver operating characteristics curve (ROC) analysis of the admission level of kidney damage markers to predict ongoing or future severe acute kidney injury (AKI). The first samples obtained from HFRS patients without AKI 2/3 for P-creatinine (5.A.), P-urea (5.B.), P-cystatin C (5.C.), U-A1M (5.D.), U-NGAL (5.E.) and U-albumin (5.F.) were used to perform a ROC curve analysis to determine whether these kidney damage markers could predict future severe AKI (AKI stage 2 or according to KDIGO 2012) and to determine the optimal cut-off level for each marker. All kidney damage markers apart from U-NGAL and U-albumin significantly predicted ongoing or future severe AKI. AKI: Acute kidney injury; A1M: α1-microglobulin; HFRS: Hemorrhagic fever with renal syndrome; NGAL: neutrophil gelatinase-associated lipocalin; P: plasma; U: urine. Figure S3: Kidney damage markers levels stratified across HFRS patients with no SPS or SPS. HFRS patients were stratified into two groups: SPS group if the ratio between eGFR cystatin C (CAPA) vs. eGFR creatinine (LM-Rev) is below 0.7 and no SPS group if the ratio is under 0.7. The estimated mean and standard error of the kidney damage markers for each group were calculated using generalized estimating equation (GEE) and adjusted for sex and age. The timeline kinetics are shown for P-creatinine (S2. A), P-cystatin C (S2. B), P-urea (S2. C.), U-A1M (S2. D), U-NGAL (S2. E) and CRP (S2. F). Significant difference between the time point and follow-up (#: >90 days post onset of HFRS) is indicated by asterisks (*** *p* < 0.001; ***p* < 0.01; * *p* < 0.05). SPS: Shrunken pore syndrome; HFRS: Hemorrhagic fever with renal syndrome; eGFR: estimated glomerular filtration rate; CAPA: Caucasian and Asian pediatric and adult subjects: LM rev” Lund–Malmö revised formula; A1M: α1-microglobulin; NGAL: neutrophil gelatinase-associated lipocalin; CRP: C-reactive protein; P: plasma; U: urine. Table S1: Association between peak levels of kidney damage markers during HFRS ^a^.
