# Supplementary material for: Cystatin C and α-1-Microglobulin Predict Severe Acute Kidney Injury in Patients with Hemorrhagic Fever with Renal Syndrome
Source: Pathogens. 2020 Aug 18;9(8):666. doi: 10.3390/pathogens9080666 (PMC7460112; doi:10.3390/pathogens9080666)
Supplement: Supplementary file 1 [file pathogens-09-00666-s001.zip › Gustafsson et al_Supplementary Tables .docx]

Table S1. Association between peak levels of kidney damage markers during HFRS ^a^

|  | **U- A1M**  (μg/mmol creatinine) | |  | **U-NGAL**  (ng/mmol creatinine) | |  | **P-Cystatin C**  (mg/ml) | |
| --- | --- | --- | --- | --- | --- | --- | --- | --- |
|  | **β** | **p-value** |  | **β** | **p-value** |  | **β** | **p-value** |
|  |  |  |  |  |  |  |  |  |
| **P-Creatinine** (μmol/l) | **15** | **0.001** |  | **1.3** | **0.01** |  | **177** | **< 0.001** |
| **P-Urea** (nmol/l) | **0.6** | **<0.001** |  | **0.06** | **0.001** |  | **6.8** | **< 0.001** |
|  |  |  |  |  |  |  |  |  |
| ^a^ The peak level of kidney damage markers obtained within samples ≤ 30 days post disease onset are included. The estimated β-coefficients were calculated using the generalized linear model and analysis for linear regression performed. The shown β-coefficients are adjusted for sex and age, and shown with the corresponding P value. This corresponds to the change in the level of kidney dysfunction markers for 1 unit increase for kidney damage markers (U-A1M, U-NGAL and P-NGAL; slope). Significant associations are shown highlighted in bold.  A1M: α1-microglobulin; HFRS: Hemorrhagic fever with renal syndrome; NGAL: neutrophil gelatinase-associated lipocalin; P: plasma; U: urine. | | | | | | | | |
