# Supplementary material for: Cystatin C and α-1-Microglobulin Predict Severe Acute Kidney Injury in Patients with Hemorrhagic Fever with Renal Syndrome
Source: Pathogens. 2020 Aug 18;9(8):666. doi: 10.3390/pathogens9080666 (PMC7460112; doi:10.3390/pathogens9080666)
Supplement: Supplementary file 1 [file pathogens-09-00666-s001.zip › Gustafsson et al_Supplementary figures.pptx]

## Slide 1
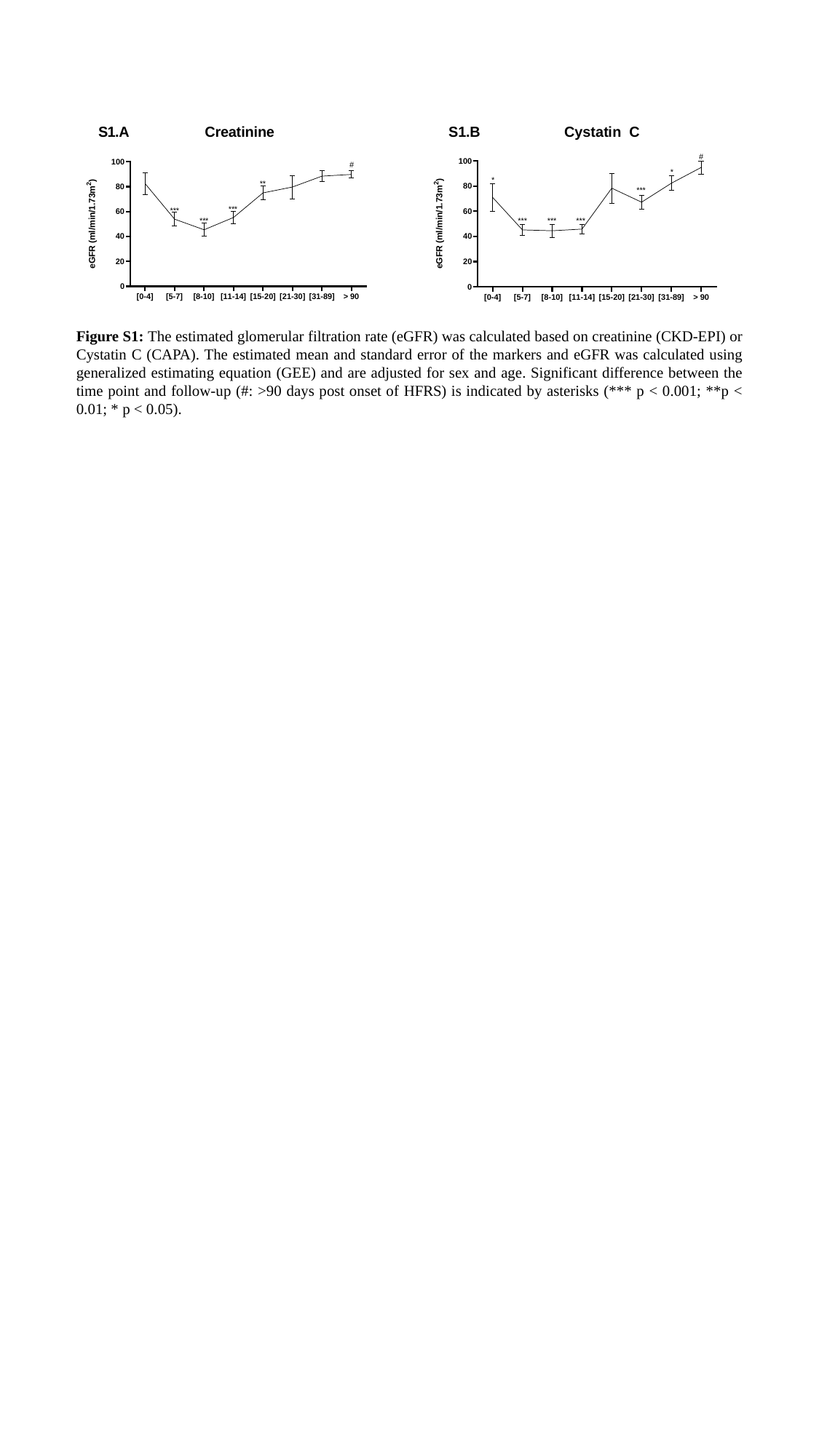

Figure S1: The estimated glomerular filtration rate (eGFR) was calculated based on creatinine (CKD-EPI) or Cystatin C (CAPA). The estimated mean and standard error of the markers and eGFR was calculated using generalized estimating equation (GEE) and are adjusted for sex and age. Significant difference between the time point and follow-up (#: >90 days post onset of HFRS) is indicated by asterisks (*** p < 0.001; **p < 0.01; * p < 0.05).

## Slide 2
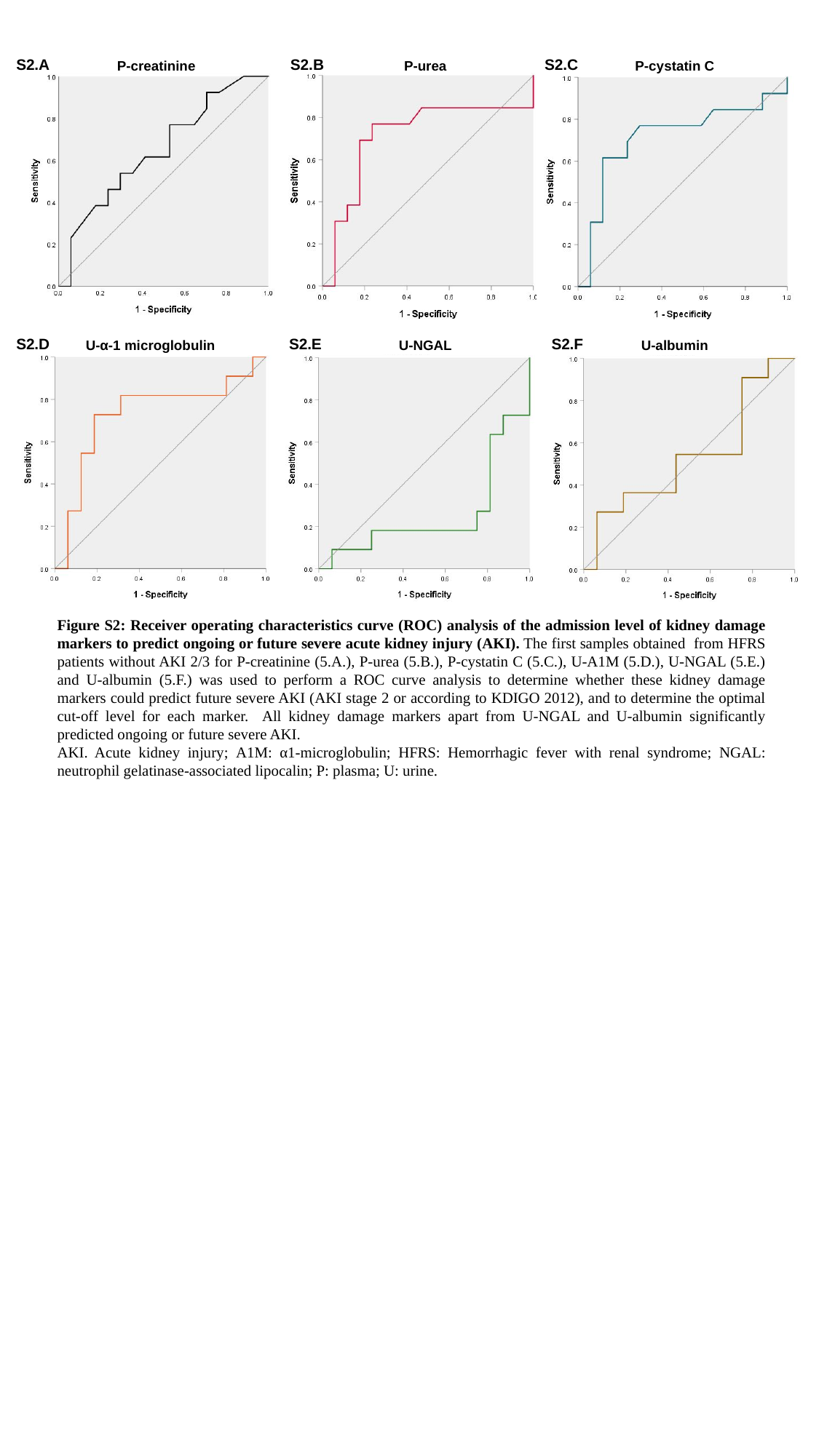

S2.A
S2.B
S2.C
P-creatinine
P-urea
P-cystatin C
S2.D
S2.E
S2.F
U-α-1 microglobulin
U-NGAL
U-albumin
Figure S2: Receiver operating characteristics curve (ROC) analysis of the admission level of kidney damage markers to predict ongoing or future severe acute kidney injury (AKI). The first samples obtained from HFRS patients without AKI 2/3 for P-creatinine (5.A.), P-urea (5.B.), P-cystatin C (5.C.), U-A1M (5.D.), U-NGAL (5.E.) and U-albumin (5.F.) was used to perform a ROC curve analysis to determine whether these kidney damage markers could predict future severe AKI (AKI stage 2 or according to KDIGO 2012), and to determine the optimal cut-off level for each marker. All kidney damage markers apart from U-NGAL and U-albumin significantly predicted ongoing or future severe AKI.
AKI. Acute kidney injury; A1M: α1-microglobulin; HFRS: Hemorrhagic fever with renal syndrome; NGAL: neutrophil gelatinase-associated lipocalin; P: plasma; U: urine.

## Slide 3
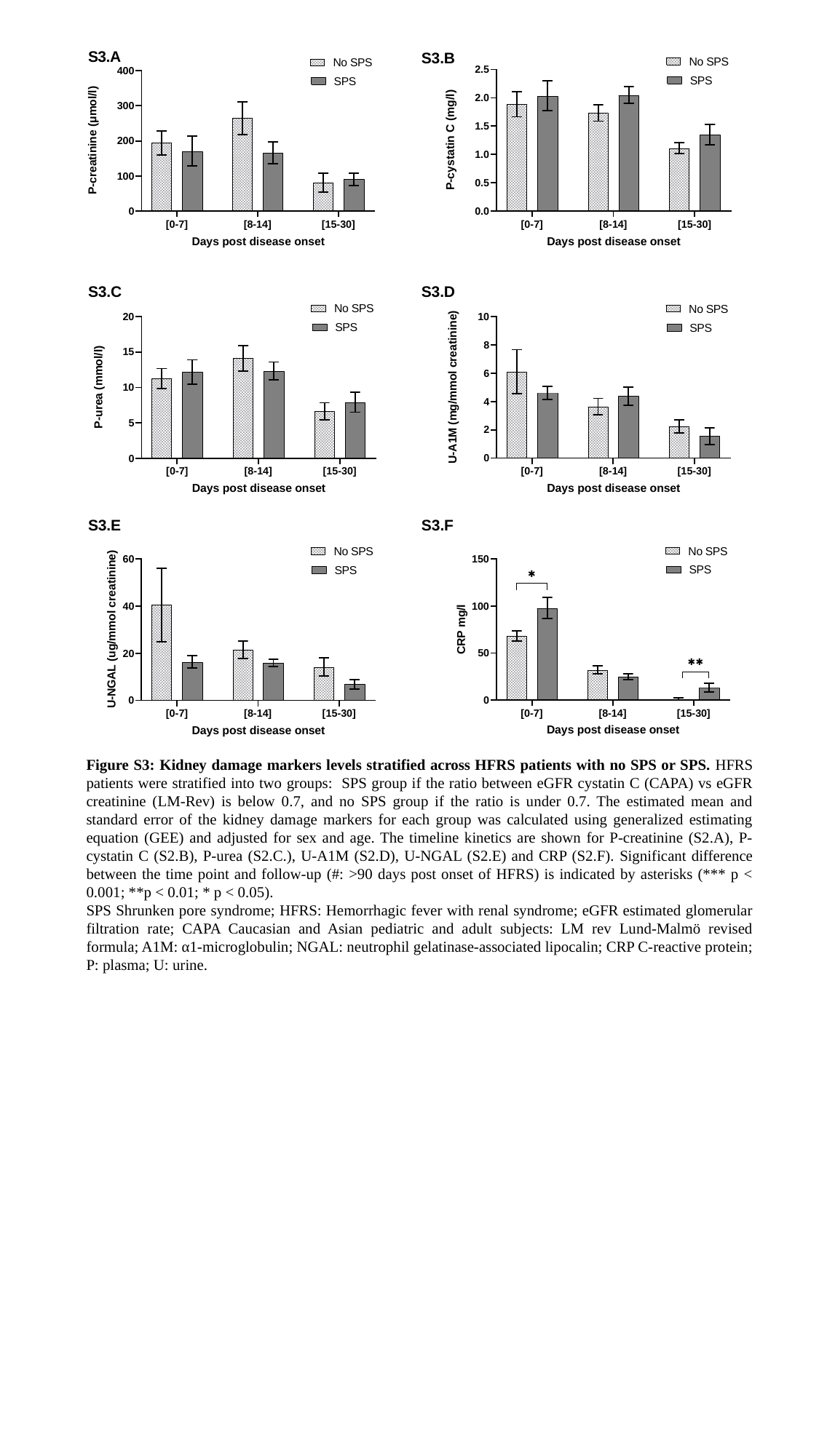

Figure S3: Kidney damage markers levels stratified across HFRS patients with no SPS or SPS. HFRS patients were stratified into two groups: SPS group if the ratio between eGFR cystatin C (CAPA) vs eGFR creatinine (LM-Rev) is below 0.7, and no SPS group if the ratio is under 0.7. The estimated mean and standard error of the kidney damage markers for each group was calculated using generalized estimating equation (GEE) and adjusted for sex and age. The timeline kinetics are shown for P-creatinine (S2.A), P-cystatin C (S2.B), P-urea (S2.C.), U-A1M (S2.D), U-NGAL (S2.E) and CRP (S2.F). Significant difference between the time point and follow-up (#: >90 days post onset of HFRS) is indicated by asterisks (*** p < 0.001; **p < 0.01; * p < 0.05).
SPS Shrunken pore syndrome; HFRS: Hemorrhagic fever with renal syndrome; eGFR estimated glomerular filtration rate; CAPA Caucasian and Asian pediatric and adult subjects: LM rev Lund-Malmö revised formula; A1M: α1-microglobulin; NGAL: neutrophil gelatinase-associated lipocalin; CRP C-reactive protein; P: plasma; U: urine.
